# Supplementary material for: Go west: Population genomics reveals unexpected population fluctuations and little gene flow in Western hemisphere populations of the predatory lady beetle, Hippodamia convergens
Source: Evol Appl. 2023 Dec 29;17(1):e13631. doi: 10.1111/eva.13631 (PMC10810170; doi:10.1111/eva.13631)
Supplement: Supplementary file 1 — Appendix S1. [file EVA-17-e13631-s001.docx]

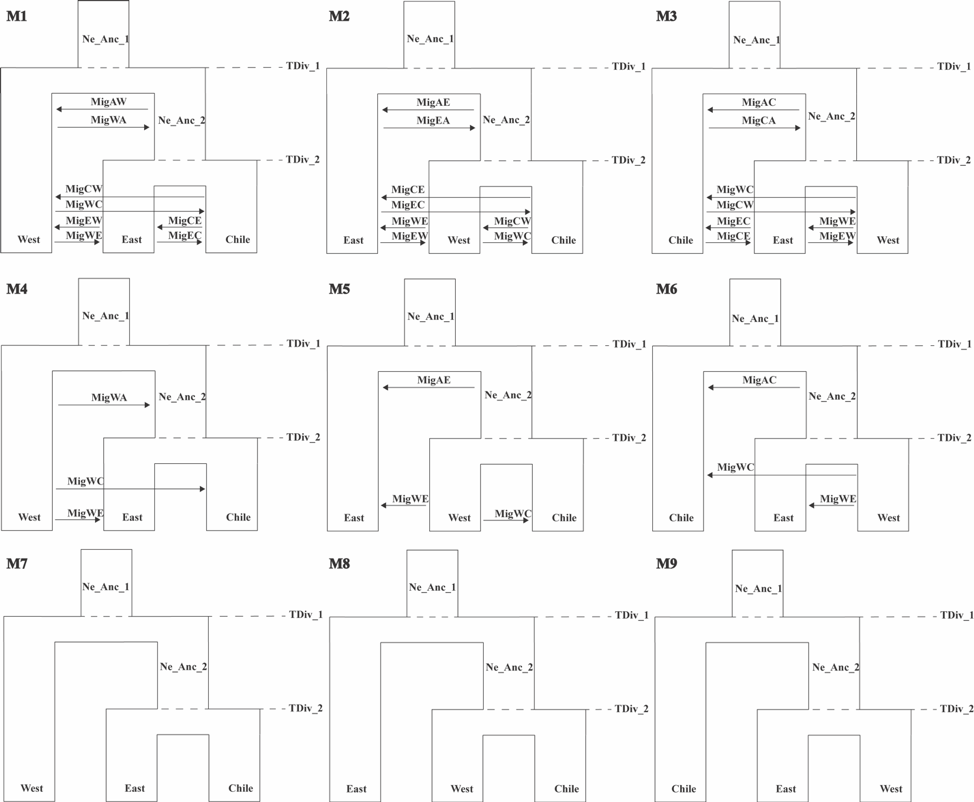


Figure S1: All evolutionary models and competing topologies tested in FSC2 under the three population model as estimated by ADMIXTURE.


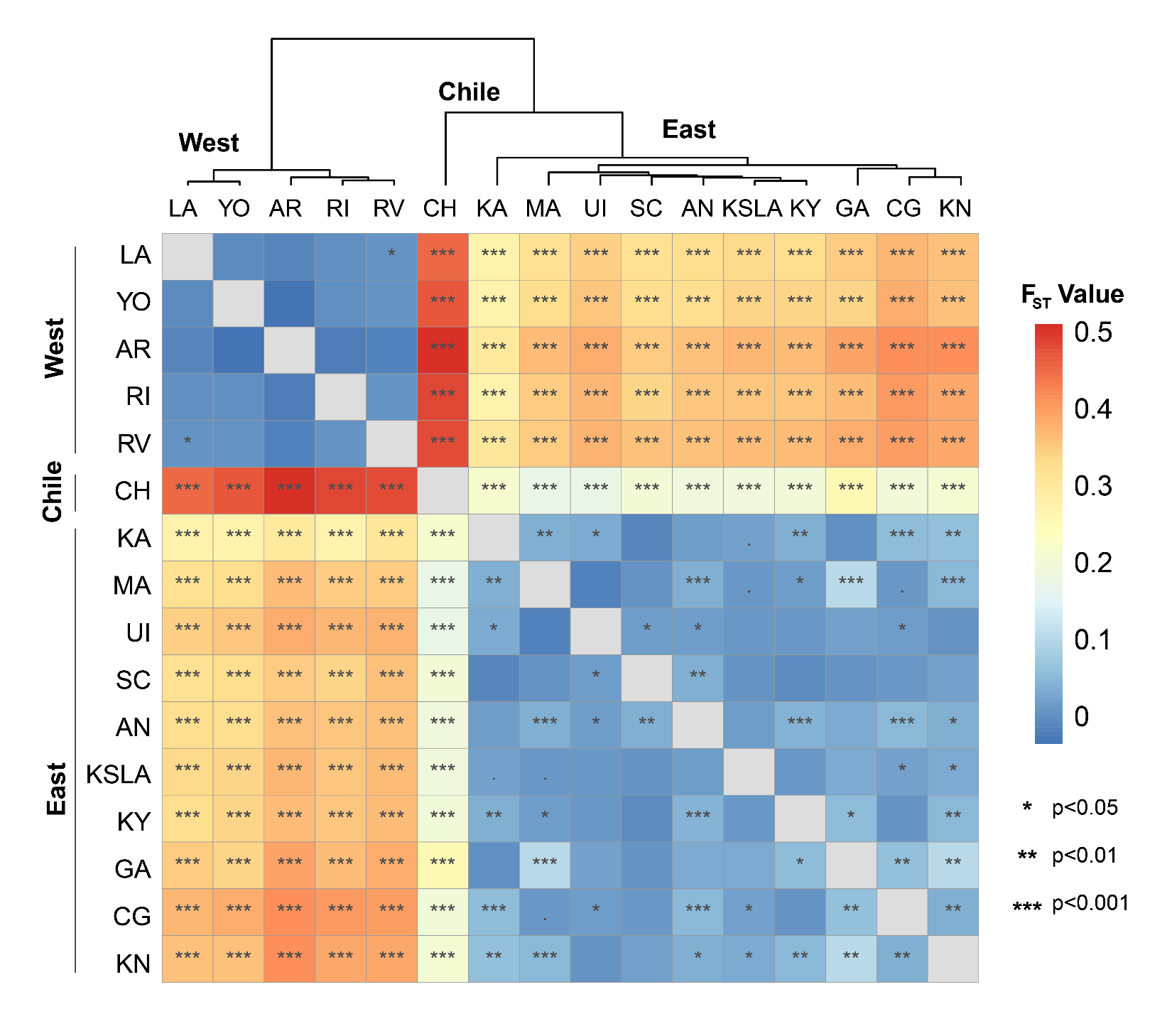


Figure S2: Estimates of pairwise Weir and Cockerham’s Fst across all sampled *H. convergens* populations.


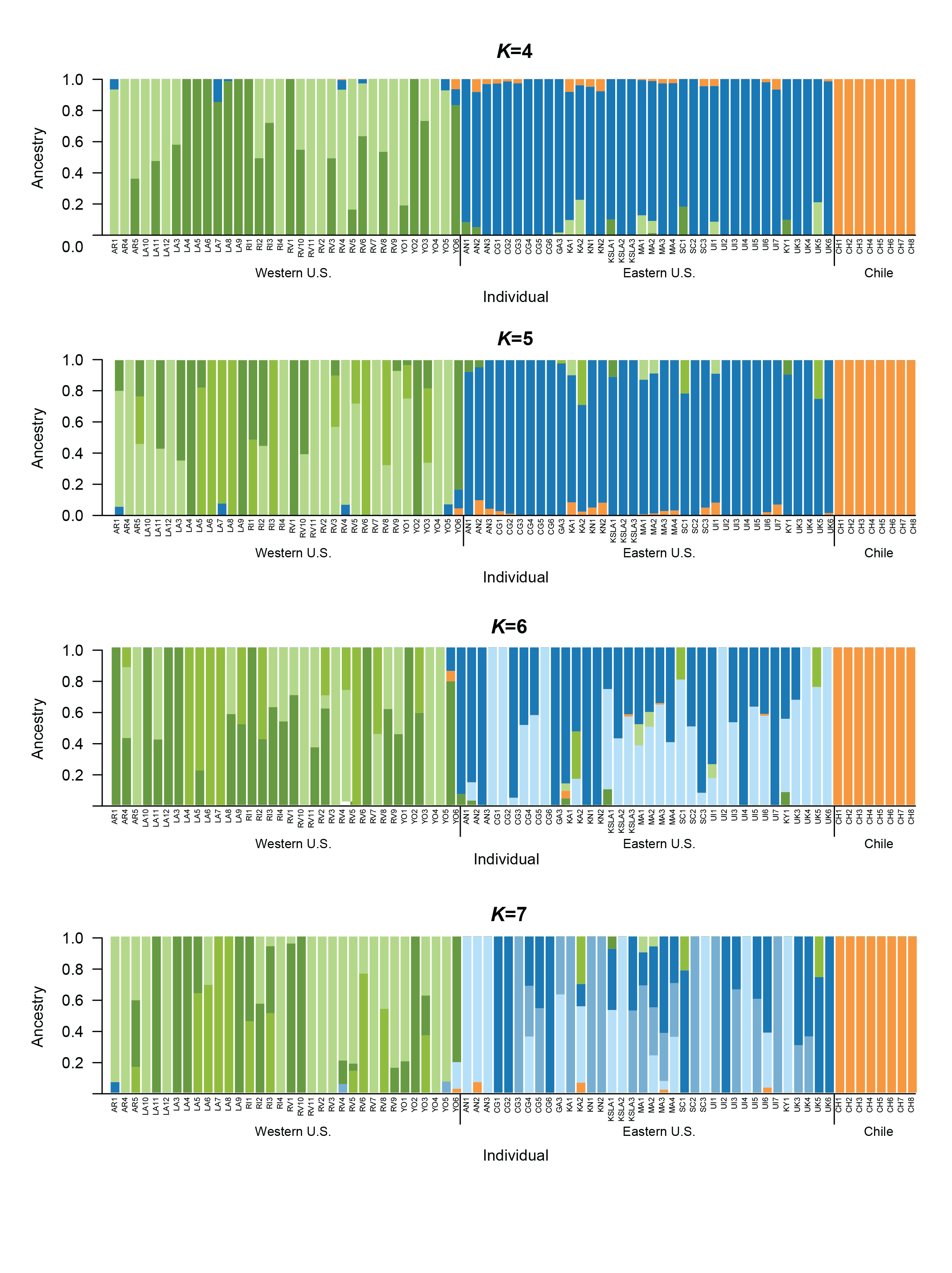


Figure S4: Ancestry proportions plotted across all sequenced *H. convergens*, as estimated by ADMIXTURE (K=4,5,6,7)

Table S1. Sampling localities, GPS Coordinates, and collection information for *H. convergens* individuals. Note that GPS coordinates for insectary-collected beetles are approximations.

| **Individual**  **Symbol** | **Location** | **Lat** | **Long** | **Date Collected** | **# of Samples Sequenced** | **# of Samples Dropped** |
| --- | --- | --- | --- | --- | --- | --- |
| AR | Arbico Organic in Tucson, AZ | 32.45°N | 110.79°W | May 2011 | 6 | 3 |
| LA | Angeles National Forest, Los Angeles City, CA | 34.44°N | 118.11°W | Oct 2011 | 10 |  |
| RI | San Jacinto Mountain Reserve, Riverside City, CA | 33.81°N | 116.68°W | Oct 2011 | 4 |  |
| RV | Rincon-Vitova Insectary in Ventura, CA | 34.29°N | 119.29°W | Oct 2015 | 12 |  |
| YO | Yolo County, CA | 38.7°N | 121.87°W | Apr 2012 | 6 |  |
| AN | Ankeny, Iowa | 41.74°N | 93.6°W | May 1992 | 3 |  |
| CG | Coffee County, GA | 31.56°N | 82.84°W | Aug 2011 | 6 |  |
| GA | Tifton, GA | 31.45°N | 83.52°W | Apr 1994 | 1 |  |
| KA | Kadoka, SD | 43.84°N | 101.51°W | Jun 1993 | 2 |  |
| KN | Knoxville, Iowa | 41.32°N | 93.11°W | May 1992 | 2 |  |
| KSLA | Lawrence, Kansas | 36.97 N | 95.23 W | May 2015 | 3 |  |
| KY* | UK North Farm | 38.14 N | 84.51 W | May 2015 | 1 |  |
| MA | Manhattan, Kansas | 39.19°N | 96.57°W | May 2011 | 11 | 7 |
| MC | Mitchell County, Iowa | 43.36 N | 92.77 W | Unknown | 1 |  |
| MUN | Muncie, Indiana | 40.19 N | 85.40 W | May 2012 | 1 |  |
| SC | Tall Grass Prairie Site, Strong City Kansas | 38.39 N | 96.53 W | May 2015 | 3 |  |
| UI | Urbandale, Iowa | 41.63°N | 93.71°W | Oct 2015 | 7 |  |
| UK* | UK North Farm, KY | 38.14°N | 84.51°W | Oct 2011 | 4 |  |
| CH | Santiago, Chile | 33.45°S | 70.67°W | Nov 2011 | 8 |  |

*Samples pooled for all analyses

Table S2. Illumina sequence reads for each individual.

| Individual | Total Reads | No RadTag | Low Quality | Reads Retained |
| --- | --- | --- | --- | --- |
| 1c | 2995968 | 15026 | 14385 | 2966557 |
| 1cA | 4900528 | 11214 | 26659 | 4862655 |
| **2c** | 409710 | 8143 | 2352 | 399215 |
| 3c | 1322626 | 7005 | 6232 | 1309389 |
| 3cA | 3990640 | 9316 | 18430 | 3962894 |
| 4c | 838298 | 7569 | 3830 | 826899 |
| 4cA | 1481192 | 7577 | 6916 | 1466699 |
| 5c | 2796856 | 12546 | 13442 | 2770868 |
| 5cA | 4924756 | 14518 | 24399 | 4885839 |
| AN1 | 3391414 | 5936 | 15768 | 3369710 |
| AN2 | 2980832 | 5104 | 14058 | 2961670 |
| AN3 | 3166168 | 17209 | 14974 | 3133985 |
| **AR1** | 827064 | 5875 | 4061 | 817128 |
| **AR2** | 485126 | 4156 | 2688 | 478282 |
| **AR3** | 271230 | 3749 | 1503 | 265978 |
| AR4 | 1155104 | 4703 | 6518 | 1143883 |
| AR5 | 2002780 | 8913 | 11761 | 1982106 |
| CG1 | 2447188 | 5931 | 12285 | 2428972 |
| CG2 | 2919260 | 7477 | 15023 | 2896760 |
| CG3 | 4336964 | 18026 | 21855 | 4297083 |
| CG4 | 4324316 | 12041 | 20917 | 4291358 |
| CG5 | 3968298 | 11291 | 17045 | 3939962 |
| CG6 | 2944178 | 9473 | 13610 | 2921095 |
| CH1 | 4169932 | 12664 | 20263 | 4137005 |
| CH2 | 5284496 | 9539 | 24405 | 5250552 |
| CH3 | 1918404 | 19203 | 10024 | 1889177 |
| CH4 | 2191154 | 9547 | 10450 | 2171157 |
| CH5 | 2332586 | 8261 | 11598 | 2312727 |
| CH6 | 1169270 | 16902 | 5227 | 1147141 |
| CH7 | 760818 | 9156 | 3747 | 747915 |
| CH8 | 690512 | 4839 | 4269 | 681404 |
| GA3 | 2334332 | 10472 | 11587 | 2312273 |
| KA1 | 1494258 | 5525 | 7374 | 1481359 |
| KA2 | 1636128 | 4215 | 8029 | 1623884 |
| KN1 | 2000726 | 19172 | 10094 | 1971460 |
| KN2 | 1595216 | 5770 | 8670 | 1580776 |
| KSLA1 | 4536300 | 25244 | 21632 | 4489424 |
| KSLA2 | 4191376 | 10986 | 19410 | 4160980 |
| KSLA3 | 4882862 | 9416 | 24239 | 4849207 |
| KY1 | 1911680 | 8857 | 10110 | 1892713 |
| LA10 | 1088236 | 8631 | 6063 | 1073542 |
| LA11 | 1193658 | 4599 | 5469 | 1183590 |
| LA12 | 1323050 | 18837 | 6262 | 1297951 |
| LA3 | 2055932 | 10258 | 9592 | 2036082 |
| LA4 | 1564552 | 14906 | 7885 | 1541761 |
| LA5 | 2604182 | 6063 | 12019 | 2586100 |
| LA6 | 3126562 | 13735 | 15818 | 3097009 |
| LA7 | 3596796 | 7825 | 17569 | 3571402 |
| LA8 | 1986974 | 6832 | 9791 | 1970351 |
| LA9 | 1251152 | 6332 | 6731 | 1238089 |
| MA1 | 754216 | 6956 | 4057 | 743203 |
| MA10 | 2665060 | 9051 | 12306 | 2643703 |
| MA2 | 1078174 | 5235 | 5374 | 1067565 |
| **MA3** | 616860 | 4890 | 2600 | 609370 |
| **MA4** | 514322 | 3917 | 3038 | 507367 |
| **MA5** | 436012 | 3987 | 3283 | 428742 |
| **MA6** | 215626 | 5578 | 1372 | 208676 |
| MA7 | 2299890 | 5754 | 11342 | 2282794 |
| MA8 | 2248204 | 14240 | 10771 | 2223193 |
| MA9 | 2460212 | 9111 | 12060 | 2439041 |
| RI1 | 2526660 | 9284 | 14300 | 2503076 |
| RI2 | 2153790 | 6611 | 9397 | 2137782 |
| RI3 | 3107266 | 20771 | 15628 | 3070867 |
| RI4 | 3564984 | 10710 | 17684 | 3536590 |
| RV1 | 2308182 | 7433 | 11350 | 2289399 |
| RV10 | 3714426 | 6674 | 16372 | 3691380 |
| RV11 | 2384022 | 7732 | 11612 | 2364678 |
| RV2 | 2999400 | 7492 | 14890 | 2977018 |
| RV3 | 1954652 | 5074 | 10554 | 1939024 |
| RV4 | 1674432 | 11605 | 8097 | 1654730 |
| RV5 | 3310764 | 9219 | 15437 | 3286108 |
| RV6 | 1393418 | 5985 | 6769 | 1380664 |
| RV7 | 2308926 | 7044 | 11112 | 2290770 |
| RV8 | 3286036 | 6608 | 16453 | 3262975 |
| RV9 | 3991772 | 6476 | 20465 | 3964831 |
| SC1 | 2277802 | 19421 | 10590 | 2247791 |
| SC2 | 4340706 | 10942 | 24803 | 4304961 |
| SC3 | 4713502 | 7602 | 23854 | 4682046 |
| UI1 | 2015532 | 7586 | 9519 | 1998427 |
| UI2 | 1585724 | 19167 | 7867 | 1558690 |
| UI3 | 1542580 | 5621 | 7628 | 1529331 |
| UI4 | 1378732 | 5529 | 6764 | 1366439 |
| UI5 | 1001040 | 5139 | 4811 | 991090 |
| UI6 | 1357690 | 6206 | 6523 | 1344961 |
| UI7 | 1449640 | 6553 | 6875 | 1436212 |
| UK3 | 1230242 | 30961 | 6232 | 1193049 |
| UK4 | 2891826 | 6663 | 13152 | 2872011 |
| UK5 | 1981814 | 6934 | 9538 | 1965342 |
| UK6 | 1951562 | 8059 | 9314 | 1934189 |
| YO1 | 1406014 | 13654 | 7250 | 1385110 |
| YO2 | 1644546 | 13515 | 7606 | 1623425 |
| YO3 | 1016842 | 6998 | 5101 | 1004743 |
| YO4 | 1161226 | 18426 | 5070 | 1137730 |
| YO5 | 1635688 | 7667 | 7587 | 1620434 |
| YO6 | 2554342 | 7977 | 12472 | 2533893 |

Individuals in bold excluded from analysis due to excessive missing genotypes

Table S3. Pairwise F_ST_ values as calculated by STACKS (above the diagonal) and Arlequin (below the diagonal) for 17 populations of *H. convergens*. Sample sizes are indicated in parentheses, also indicating the lack of power for the GA, KY, MUN populations to assess significant differentiation due to small sample sizes. Absolute values differ because of differences in how the programs treat missing data and nonpolymorphic sites. Significance was determined with 10,000 permutations in Arlequin, values in bold are significant at the 0.05 level.

|  | AR | LA | RI | RV | YO | AN | CG | GA | KA | KN | KSLA | KY | MA | MUN | SC | UI | CH |
| --- | --- | --- | --- | --- | --- | --- | --- | --- | --- | --- | --- | --- | --- | --- | --- | --- | --- |
| AR (3) | - | 0.075 | 0.141 | 0.068 | 0.106 | 0.223 | 0.175 | 0.342 | 0.261 | 0.292 | 0.221 | 0.178 | 0.144 | 0.301 | 0.226 | 0.152 | 0.237 |
| LA (10) | 0.05 | - | 0.072 | 0.044 | 0.063 | 0.128 | 0.119 | 0.159 | 0.129 | 0.141 | 0.131 | 0.113 | 0.099 | 0.145 | 0.133 | 0.103 | 0.155 |
| RI (4) | 0.052 | 0.009 | - | 0.065 | 0.104 | 0.219 | 0.177 | 0.326 | 0.251 | 0.268 | 0.224 | 0.181 | 0.146 | 0.292 | 0.226 | 0.155 | 0.241 |
| RV (12) | 0.027 | 0.013 | 0.018 | - | 0.056 | 0.124 | 0.116 | 0.152 | 0.124 | 0.137 | 0.125 | 0.115 | 0.099 | 0.133 | 0.129 | 0.104 | 0.153 |
| YO (6) | 0.029 | 0.004 | 0.011 | -0.001 | - | 0.177 | 0.15 | 0.241 | 0.188 | 0.208 | 0.179 | 0.149 | 0.126 | 0.211 | 0.183 | 0.13 | 0.195 |
| AN (3 | 0.315 | **0.329** | **0.392** | **0.335** | **0.325** | - | 0.111 | 0.256 | 0.198 | 0.211 | 0.157 | 0.123 | 0.096 | 0.248 | 0.165 | 0.096 | 0.169 |
| CG (6) | **0.352** | **0.366** | **0.418** | **0.376** | **0.369** | 0.035 | - | 0.158 | 0.135 | 0.136 | 0.11 | 0.09 | 0.075 | 0.15 | 0.112 | 0.077 | 0.132 |
| GA (1) | 0.331 | 0.373 | 0.419 | 0.374 | 0.36 | 0.101 | 0.096 | - | 0.406 | 0.414 | 0.259 | 0.176 | 0.139 | 0.542 | 0.258 | 0.141 | 0.235 |
| KA (2) | 0.199 | **0.232** | 0.278 | **0.242** | **0.232** | 0.017 | 0.049 | 0.055 | - | 0.307 | 0.21 | 0.145 | 0.111 | 0.383 | 0.199 | 0.112 | 0.199 |
| KN (2) | 0.354 | **0.374** | 0.414 | 0.379 | **0.372** | 0.109 | 0.055 | 0.163 | 0.06 | - | 0.21 | 0.154 | 0.116 | 0.392 | 0.222 | 0.119 | 0.205 |
| KSLA (3) | 0.292 | **0.32** | **0.377** | **0.329** | **0.316** | 0.043 | 0.029 | 0.077 | -0.013 | 0.077 | - | 0.119 | 0.092 | 0.243 | 0.158 | 0.094 | 0.171 |
| KY (5) | **0.313** | **0.331** | **0.368** | **0.339** | **0.324** | 0.052 | 0.003 | 0.091 | 0.013 | 0.04 | 0.034 | - | 0.081 | 0.159 | 0.115 | 0.082 | 0.144 |
| MA (4) | **0.269** | **0.305** | **0.343** | **0.311** | **0.3** | 0.012 | 0.021 | 0.073 | -0.011 | 0.048 | -0.003 | 0.025 | - | 0.127 | 0.097 | 0.065 | 0.121 |
| MUN (1) | 0.244 | 0.288 | 0.335 | 0.287 | 0.279 | 0.065 | 0.057 | 0.103 | -0.033 | 0.069 | 0.01 | 0.007 | -0.011 | - | 0.246 | 0.123 | 0.22 |
| SC (3) | 0.291 | **0.308** | **0.345** | **0.319** | **0.301** | 0.047 | 0.035 | 0.081 | 0.017 | 0.075 | 0.028 | 0.004 | 0.017 | 0.041 | - | 0.099 | 0.183 |
| UI (7) | **0.312** | **0.335** | **0.382** | **0.344** | **0.334** | 0.006 | 0.008 | 0.075 | 0.012 | 0.046 | 0.014 | 0.029 | 0.004 | -0.007 | 0.045 | - | 0.121 |
| CH (8) | **0.434** | **0.424** | **0.492** | **0.425** | **0.435** | **0.147** | **0.165** | 0.281 | **0.148** | **0.226** | **0.176** | **0.162** | **0.137** | **0.187** | **0.199** | **0.135** | - |

Figure S5: Cross validation errors from ADMIXTURE analyses performed with all 9824 SNPs (unfiltered), across K = 1 to 20, indicating support for K=2 or K=3 as the "optimal" number of subpopulations across all sampled *H. convergens*

Figure S6: Ancestry proportions plotted across all sequenced *H. convergens*, as estimated by ADMIXTURE (K=2,3) at all 9824 SNPs.

Eastern USA Western USA Chile

Figure S7: Population structure of *H. convergens*, shown as a scatter plot of *H. convergens* genotypes generated by DAPC at K = 3, using all 9824 SNPs (unfiltered). E = Eastern, W = Western, and C = Chilean populations.


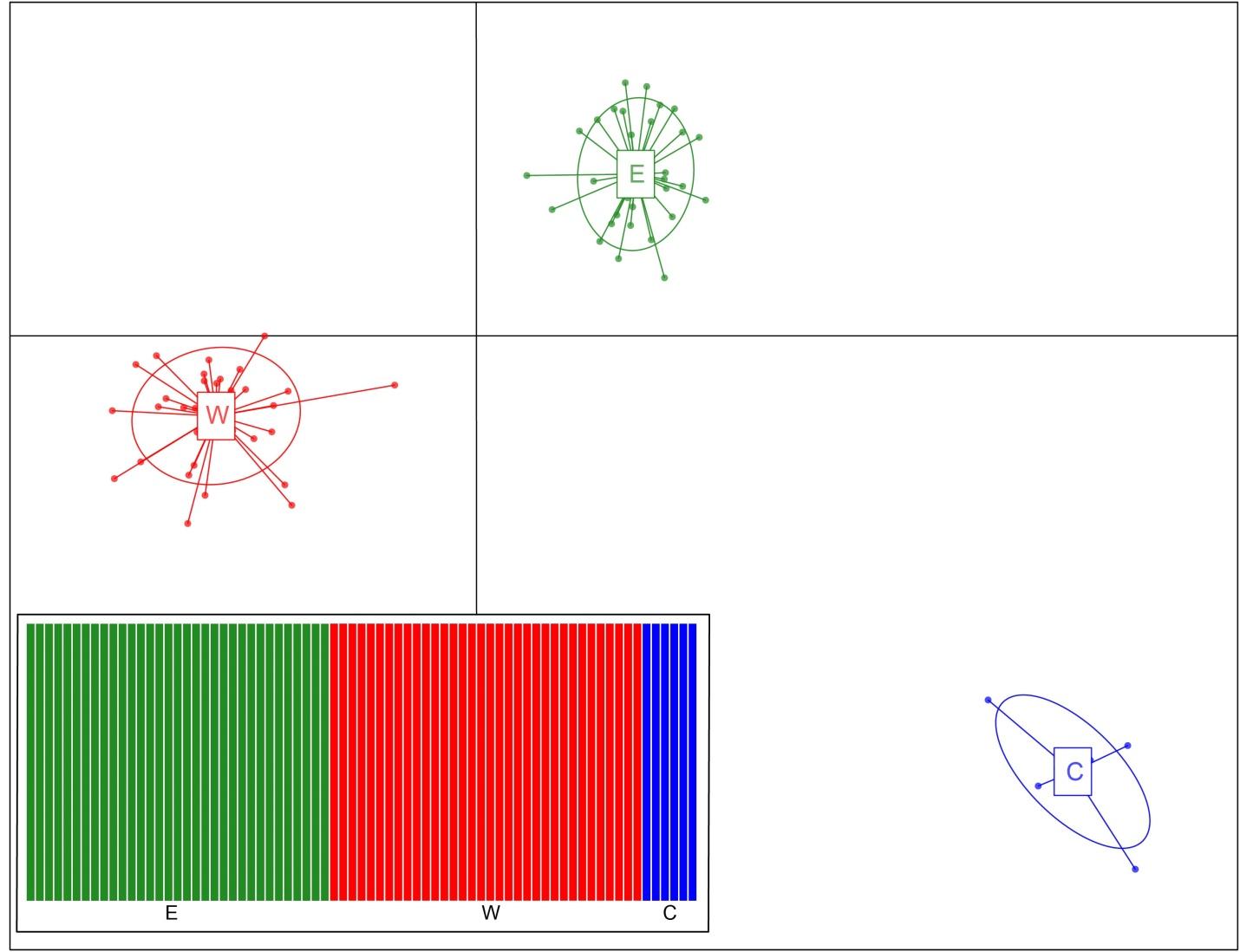


Figure S8: Cross validation errors from ADMIXTURE analyses performed after removing 8 individuals that were sampled in the 1990's (GA, KA, KN, AN, MC), indicating support for K=2 or K=3 as the "optimal" number of subpopulations across all sampled *H. convergens*

Figure S9: Ancestry proportions plotted across all sequenced *H. convergens*, as estimated by ADMIXTURE (K=2,3,4) after removing 8 individuals that were sampled in the 1990's (GA, KA, KN, AN, MC), recapitulating the Western, Eastern, Chilean split across all sampled individuals.

Figure S10: Population structure of *H. convergens*, shown as a scatter plot of *H. convergens* genotypes generated by DAPC at K = 3, after removing 8 individuals that were sampled in the 1990's (GA, KA, KN, AN, MC), recapitulating the Western, Eastern, Chilean split across all sampled individuals.


Figure S11: Plot of differentiation between (measured as Weir and Cockerham's Fst) versus observed heterozygosity within subpopulations (Western, Eastern, and Chilean) of *H. convergens*. OutFLANK analyses indicate no significant outlier locus across all loci analyzed.
